# Clinical and cost effectiveness of mechanical support for severe ankle sprains: design of a randomised controlled trial in the emergency department [ISRCTN 37807450]

**DOI:** 10.1186/1471-2474-6-1

**Published:** 2005-01-13

**Authors:** SE Lamb, RA Nakash, EJ Withers, M Clark, JL Marsh, S Wilson, JL Hutton, A Szczepura, JR Dale, MW Cooke, the Collaborative Ankle Support Trial research team (CAST)

**Affiliations:** 1 Centre for Primary Health Care Studies, Warwick Medical School, University of Warwick, Coventry, CV4 7AL, UK; 2 Kadoorie Critical Care Research Centre, John Radcliffe Hospital, Oxford, OX3 9DU, UK; 3 Department of Statistics, University of Warwick, Coventry, CV4 7AL, UK; 4 Department of Primary Care and General Practice, Medical School, University of Birmingham, Edgbaston, Birmingham, B15 2TT, UK

## Abstract

**Background:**

The optimal management for severe sprains (Grades II and III) of the lateral ligament complex of the ankle is unclear. The aims of this randomised controlled trial are to estimate (1) the clinical effectiveness of three methods of providing mechanical support to the ankle (below knee cast, Aircast^® ^brace and Bledsoe^® ^boot) in comparison to Tubigrip^®^, and (2) to compare the cost of each strategy, including subsequent health care costs.

**Methods/design:**

Six hundred and fifty people with a diagnosis of severe sprain are being identified through emergency departments. The study has been designed to complement routine practice in the emergency setting. Outcomes are recovery of mobility (primary outcome) and usual activity, residual symptoms and need for further medical, rehabilitation or surgical treatment. Parallel economic and qualitative studies are being conducted to aid interpretation of the results and to evaluate the cost-effectiveness of the interventions.

**Discussion:**

This paper highlights the design, methods and operational aspects of a clinical trial of acute injury management in the emergency department.

## Background

Sprains of the lateral ligaments of the ankle joint account for between 3 and 5% of all emergency department (ED) attendances in the UK [1], with approximately 5600 injuries each day [2]. The injury is painful and incapacitating, and, for all but the most minor injuries, weight bearing is difficult to tolerate. Activities of daily living can be significantly compromised in the first few weeks, and although acute symptoms resolve, persistent symptoms are reported to occur in between 30 to 50% of people [3-6]. Persistent symptoms include recurrent sprains, instability, swelling, unsightly appearance and pain. Crichton has developed a classification of severity of ankle injuries [7].

• GRADE I – the ligament is stretched but not torn and the anterior talofibular ligament is usually involved. The anterior draw test is negative.

• GRADE II – the ligaments are partially torn; laxity may be present and there is moderate swelling.

• GRADE III – complete rupture of the ligament resulting in joint instability. The anterior draw test is positive.

The focus of this trial is Grade II and III sprains (referred to as severe sprains from this point). Severe sprains have poorer prognosis, and necessitate more intensive management than Grade I sprains (minor sprains). Minor sprains are considered to be self-limiting and require minimal treatment.

Recent systematic reviews have highlighted the lack of high quality evidence to support clinicians in managing severe sprains [2,7]. There are few reliable studies describing long-term outcome. Most trials required x-ray evidence of talar tilt or an arthrogram for inclusion and are, therefore, not generalisable to clinical practice in the ED. Current practice ranges from no intervention, physiotherapy, and different types of mechanical support to surgical repair of the ligaments. A recent UK survey of 83 EDs demonstrated the most popular treatments were ice, elevation, Tubigrip^® ^support stocking and exercise, each of which was reported as being used in most cases by over 70% of respondents [8]. In addition, over half of responding departments reported that crutches, early weight bearing, and non-steroidal anti-inflammatory drugs were used in most cases. Follow up was used only in selected cases.

Practice has been gradually changing from immobilisation of the injured joint to early mobilisation. To aid mobilisation the use of mechanical supports has been suggested. These supports vary in the amount of ankle movement they allow, but all encourage ankle flexion/extension and aim to minimise inversion/eversion that theoretically reduces the risk of further ligament injury. Movement is also proposed as a way of retraining the ankle proprioception which may reduce recurrent injury rate [9]. Other hypothesised benefits of mechanical supports are early restoration of functional mobility, rapid return to usual activities, reduction of pain because the joint is stabilised and protected, and improved quality of life. However, there is also the possibility of discomfort and inconvenience owing to restriction of joint range, and delayed healing, skin and circulatory problems.

The present study has two aims

1. To estimate the clinical effectiveness of three different methods of mechanical support (below knee cast, Aircast^® ^ankle support and Bledsoe^® ^boot) in comparison to Tubigrip^® ^in

a. The recovery of mobility (primary outcome)

b. The recovery of usual occupation

c. Avoidance of residual symptoms including recurrent instability, lasting limitation of physical activity, and need for further medical, rehabilitation or surgical treatment.

2. To measure the cost of each strategy, including treatment and subsequent health care costs.

The NHS National Co-ordinating Centre for Health Technology Assessment has guided the selection of treatments. Tubigrip^® ^has been chosen as the reference treatment because it is the cheapest and one of the most commonly used [10]. The Bledsoe^® ^boot is lightweight and incorporates a novel design to promote ease of walking. However it is considerably more expensive, and it's clinical and cost effectiveness is yet to be proven. The below knee cast is Scotch^® ^Cast is commonly used for casting in the NHS [9]. There is a range of lightweight mechanical supports available, and we have selected the Aircast^® ^Brace.

## Methods

**Figure 1:**
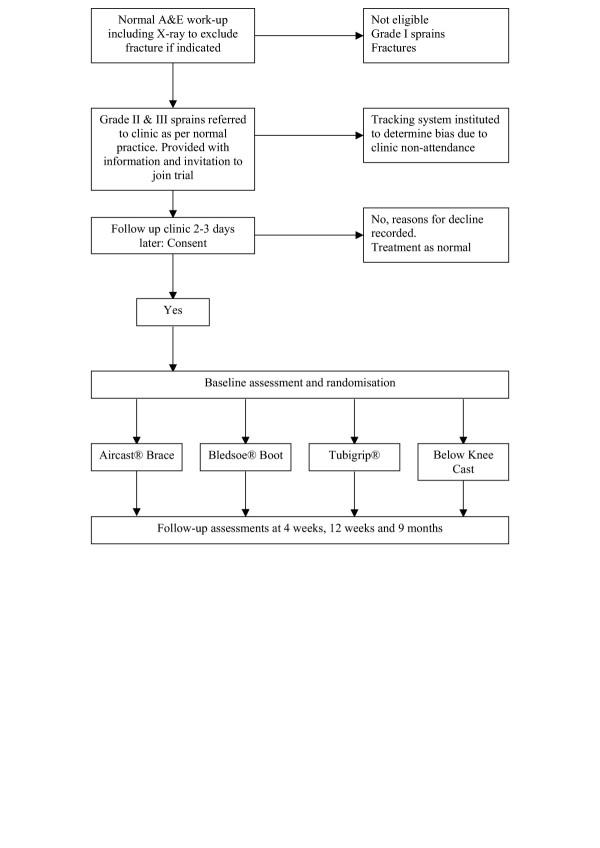
Study design

This pragmatic randomised controlled trial is being run in 6 trusts (covering eight hospitals) across the UK. The trusts are: Birmingham Heartlands and Solihull NHS Trust, North Bristol NHS Trust, Oxford Radcliffe Hospitals NHS Trust, South Warwickshire General Hospitals NHS Trust, University Hospitals Coventry and Warwickshire NHS Trust and the Worcestershire Acute Hospitals NHS Trust. Figure 1 provides an overview of the trial method and patient journey. Multi-centre Research Ethics Committee approval has been awarded by the Northern and Yorkshire Regional Office and local ethical and research governance approvals have been obtained. Informed consent to participate in the trial and allowing researchers to access hospital records is obtained from all participants.

### Study population

The target population is people attending the ED with a severe sprain of the lateral ligament complex of the ankle.

### Inclusion criteria

People who attend with sprain of the ankle who are unable to weight bear, aged 16 years and older, and give informed consent. Weight bearing is used as a proxy for severe sprains as clinical grading is not possible in the acute phase [7]. Flake fractures (<=2 mm) of the lateral malleolus are included as they are normally treated as soft tissue injuries [11].

### Exclusion criteria

Age less than 16 years old, ankle or other fracture sustained in addition to the sprain (such as to the wrist, head etc). An x-ray is used to exclude fracture where this is clinically indicated and as guided by the Ottawa guidelines [12]. People who can weight bear and those with fractures are ineligible for the trial, and are managed in accordance with normal local practices. Age is used as an exclusion criterion because of the complications involved in the management of epiphyseal injuries (growth plate injuries). Growth plate injuries would not normally be managed using the treatments being tested [13].

Patients are also excluded if they have a contra-indication to any of the four arms of the trial. This is most likely to occur if a patient has a DVT, high risk of DVT or other circulatory disturbance. Other contra-indications include poor skin viability preventing splinting or casting. The decision to exclude on these criteria is made by the attending clinician.

### The process of identifying participants

A standard approach to identifying potential participants has been instituted across all participating centres. The approach was designed in consultation with the departments to dovetail with current procedures, and eradicate duplication of clinical and research data collection. The attending clinician (nurse practitioner, physiotherapist or doctor) assesses weight-bearing status. These data are recorded on a form that includes details of the remainder of the clinical examination, including if indicated, x-ray results. At this stage, people able to weight bear are excluded.

The attending clinician gives a brief explanation of the trial, an information pack, and arranges an appointment at a follow-up clinic two to three days later. Delay is an accepted practice in the application of mechanical supports, allowing for the resolution of acute swelling. It also filters out patients in whom the diagnosis of severe sprain at initial contact was incorrect, and gives participants sufficient time to consider whether they wish to participate in the trial. A physiotherapist who staffs the follow-up clinic is responsible for checking eligibility and providing a further opportunity for patients to ask questions. If appropriate, the physiotherapist recruits the patients, contacts the randomisation centre, and arranges application of the treatments. Reasons for declining to participate in the trial are recorded.

### Assessing the generalisability of the results

Data on all ankle sprains attending the participating EDs during the recruitment period are collected using a standardised proforma. A copy of this proforma, which is entered into the medical record, is anonymised and passed to the research team. It provides descriptive data on the injury pattern, occupation, age, and sex of patients, including those who are ineligible or decline trial participation at various time points. Completed proformas are audited against ED attendance records to check whether attending clinicians have referred appropriate patients to the follow-up trial clinics. This allows clinicians who are unfamiliar with the trial to be identified and approached individually to be briefed on trial procedures.

### Treatment allocation

Randomisation is stratified by centre and provided via a phone-in service, which utilises a computer generated random allocation. Allocation concealment, which shields people who enter patients into a trial from knowing future allocations, is thereby ensured. The randomisation service is independently administered and quality controlled by the Birmingham Cancer Trials Unit.

### Treatment protocol

The mechanical supports are fitted to each individual by a trained health professional (physiotherapist, nurse or plaster technician) in the follow-up clinic, to ensure comfort and correct fit. Participants are provided with standardised written and verbal instructions including when to remove the support, encouragement of normal walking with limits of tolerance, simple exercise advice, what to do in the event of experiencing difficulties with the support, and washing instructions. The protocols for duration of support, weight-bearing status, and activity have been determined by the manufacturers recommendations and the results of a national survey of practice completed in the planning phase of the trial [8]. Treatments are applied within three days of the injury, and within an hour of randomisation.

### Other treatments

All other treatments are standardised and include the provision of crutches, advice to elevate and pain relieving medications if needed. Withdrawal of the latter treatments would be inappropriate as they constitute normal and accepted care. Physiotherapy is not provided as part of the trial treatment protocol, and is not part of routine care provided in the participating centres [9]. If the participant receives these types of treatment in addition to the trial protocol, this is recorded as an outcome.

### Baseline and outcome measures

Clinical status is measured at baseline, 4 weeks, 12 weeks and 9 months. The baseline assessment also includes date of birth, sex, body mass index, ethnicity, an assessment of pre-injury abilities including usual levels of mobility, engagement in sporting activity, usual occupation and employment (including hours worked and type of work undertaken) and completion of the baseline version of the outcome questionnaires.

The selection of outcome measures is based on a systematic review completed during the preparatory stage [14], and are shown in Table 1. There is a paucity of information on the psychometric properties of self-completed questionnaires for ankle conditions. The Foot and Ankle Outcome Score (FAOS) is a questionnaire that ascertains functional limitations (including mobility) and the severity of other symptoms after ligament sprains, and has evidence of validity and reliability [10]. Previous versions have been validated against objective tests of ankle function [15]. The Functional Limitations Profile (FLP) is the British version [16] of the Sickness Impact Profile [17]. We are using the FLP occupation and mobility sub-scales to provide more detailed information on the impact of the injuries and treatments including adaptations that occur after the injury. The FLP has not been used in studies of ankle injuries previously. Health related quality of life will be measured using the SF-12 version 1 and EQ-5D [18]. Return to normal occupation and leisure activities will be recorded as the date that people return to work and normal activities. Patients are asked to make a record of significant dates on a calendar to aid recall. The effectiveness of these calendars is being tested in a separate study.

**Table 1:** Outcome measures

**Outcome**	**Outcome measure**
Functional limitations and severity of symptoms after ligament sprains	Foot and Ankle Outcome Score (FAOS)
Impact of injuries and treatments including adaptations that occur after injury	Functional Limitations Profile (FLP) occupation and mobility sub scales
Health related quality of life	Short Form 12 version 1 (SF-12 v1)
Health related quality of life	EuroQol (EQ-5D)
Health economics	Resource Use Questionnaire
Return to normal occupation and leisure activities	Date of return to work and normal activities recorded on a trial calendar

### Timing of follow-up

The mechanical supports are likely to have maximal impact and benefit during the first three months of recovery, and this time period is defined as the primary time point. We anticipate that some participants will still be wearing the mechanical support at 4 weeks. The natural time course of recovery of ankle sprains is for functional limitations to stabilise between 3 and 9 months and it is expected that the difference between treatments will narrow in the longer term as the majority of people will recover [3,5,6]. Participants will be followed to 9 months to ensure that there are no longer-term complications from injury or their treatments.

### Follow-up and masking

Follow-up data are collected by postal questionnaire. We have implemented an intensive approach to follow-up. Participants are mailed a follow-up questionnaire, and if a response is not received within one week, they are telephoned to ensure they have received the questionnaire. A second questionnaire is mailed if necessary. If there is still no response, the participant is contacted one week later, and a core set of outcomes are collected over the telephone. All follow-up data are collected and analysed by individuals who are independent of the recruitment, randomisation, baseline assessment and are masked from the treatment provision. This level of masking will be maintained until final analysis of the data has been completed. The only exception to this rule will be if the data monitoring committee require unmasked data, and in this circumstance, only the independent Chairperson will be aware of assignments.

### Quality assurance

Trial procedures are audited at regular intervals to ensure compliance with the protocol. Maintaining trial profile and competence in trial procedures is challenging in the ED, because of a high turn-over of staff and a diversity in the staff groups dealing with injuries in different hospitals. To combat these difficulties, training is provided on cycles that coincide with staff rotation, as well as various other time points (e.g. in-service training).

### Data analyses

The analysis will be conducted as intention to treat. An analysis of all people who completed the trial will be undertaken, and in addition, a sensitivity analysis will be undertaken to assess the range of potential biases that could result from loss to follow up or withdrawal. Numerical and graphical summaries of all the data will be compiled, including a detailed description of missing data at the clinic visit, questionnaire and individual level.

Logistic and log-linear multinomial regression models will be used to provide estimates of the recovery rates and the prevalence of residual symptoms, with confidence intervals. As there are multiple centres and repeat assessment, random effect (or hierarchical) models will be used to investigate the components of variation. The common current and cheapest therapy is Tubigrip^®^, so each of the three other treatments will first be compared with it. Any treatments found to be more effective than Tubigrip^® ^will be compared with each other. Regression modelling will allow an assessment of factors that might indicate the appropriate choice of treatment. As these analyses are pre-specified, issues of multiple comparisons are minimal.

The sensitivity of the above analyses to missing data at various levels will be assessed and quantified using modern statistical methods for incomplete multivariate data [19].

### Economic analysis

Severe sprains may have a range of direct cost consequences across primary and secondary health care, they may also have cost consequences for patients themselves in terms of their personal expenditure and return to work. The costing study will seek to adopt a broader societal perspective, including patient costs, when estimating differences in the cost of resources used in the four arms of the trial. The economic analysis will compare resource use (costs) with any measurable changes in health outcomes (benefits). The health outcomes of the four technologies will be measured in terms of changes in specific disease parameters and validated measures of health-related quality of life (HRQL). Any uncertainties in the cost and outcomes data will be incorporated into a sensitivity analysis.

The cost of each mechanical support protocol will be determined through observation and will include staff time, overheads, equipment and transport; plus follow-up visits to hospital, GP surgeries, physiotherapists or others. The consequences for patients in terms of GP and hospital visits, including travel expenses and time off work, and expenditure on aids or private practitioner input are obtained from structured resource use questions added to patient follow-up questionnaires. Further treatments recorded include pain relieving medications, anti-inflammatory and other topical agents, bandages, supports or footwear. Patients are also asked to distinguish whether these are NHS-funded or private treatment paid for by the individual or private provider. Patient self-reported information on service use has been shown to be accurate in terms of intensity of use of different services [20]. Hospital notes and records will also be audited for information on service use. The cost of primary and hospital services will be estimated from a variety of sources, including the finance departments of the trial hospitals and services concerned and national sources [21,22]. Differences in resource use and outcomes are ascertained in follow-up questionnaires at 12 weeks and 9 months.

The appropriate technique of economic evaluation will depend on the results of the study [23]. The simplest eventuality would be where the least expensive intervention is found to be better on at least one outcome measure and no worse on any other i.e. dominant. Another is where two interventions have the same outcomes in which case cost-minimisation analysis will be used to compare the two. However, where an intervention is clearly better in terms of outcome but is also more costly, a different approach is required. One accepted method is to compare the different interventions in terms a single outcome measure identified as clinically important for the condition being treated. The primary outcome meets this requirement. Therefore, the costs per unit improvement in mobility will be used to provide an estimate of overall cost-effectiveness; average and incremental (relative to reference treatment) cost-effectiveness ratios will be estimate for the different treatments used. However, this approach does not allow comparison with other types of intervention/condition combinations and also does not consider the value (utility) of differences in improvement in health status. In the present study, the EQ-5D will be used to generate such utility scores that can be compared to costs. A cost-utility analysis will estimate outcomes in terms of differences in quality-adjusted life years (QALYs), representing the period of life subsequent to a health care intervention weighted or adjusted for the quality of life experienced by the patient during that period, and compare these with cost for the four interventions [23]. The cost-utility analysis will present the incremental cost of the extra benefit gained both in summary form in terms of incremental cost per QALY, and also using a 'disaggregated' approach where the extra costs are presented alongside the HRQOL dimensions such as pain.

### Sample size

Although there was a paucity of published data available to inform the sample size estimate at the outset of the trial, the preliminary phase of the study has enabled us to make a more accurate estimation of the sample size required. The presented estimate is based on a standard sample size calculation for a two-sample t-test with equal variances and a significance level of 0.05, using the variance estimated from an ANOVA of the 4-week data (n = 100). The minimal clinically important difference is set at 10 percent, which represents a small to moderate effect size. A target of 600–650 participants will allow us to detect clinically important outcomes at 4 and 12 weeks. We are powered >90% to detect differences of 10 percent in the primary outcomes, and have sufficient power to detect differences in a range of secondary outcomes at 80% power. To account for the possibility of loss to follow-up, the estimate includes an inflation of 20%. However, the trial should be able to report whether any of the treatments have a sustained negative effect on outcome at 9 months. We are taking a pragmatic approach to estimating the sample size, and the Data Monitoring Committee are reviewing assumptions underlying the calculation at 6-monthly intervals.

### Qualitative sub-study

A purposive sample of randomised patients will be interviewed to obtain qualitative data on the patients' experiences of the various treatments in a semi-structured interview. Patients will also be asked for their views on their willingness to pay a deposit for the boots/splints to encourage their return. Up to 20 patients will be interviewed using a semi-structured format. The data will be analysed using thematic content analysis.

## Conclusions

We have described the protocol and conduct of a large scale UK randomised controlled trial of mechanical supports for the management of acute severe ankle sprains. There has been a paucity of good quality research conducted to date, which may be partly explained by the challenges implicit to studying acute events within EDs [24,25]. These include a very short window of opportunity in which patients satisfy the criteria of acute injury and reliance on attending clinical staff to identify and approach for inclusion potential participants who are often in a state of discomfort. The trial is currently in the middle of the recruitment phase and is running well. Trial procedures have been well received by both patients and clinicians. Much effort has gone into maintaining the profile of the trial and disseminating trial procedures across all disciplines of ED staff (e.g. doctors, nurses, reception staff and plaster technicians). This has been paramount to the success of the trial. By referring potential patients to the follow-up trial clinics ED personnel, with no direct involvement in the trial, play a vital role in the trial process. The intense follow-up has produced good completion rates. Using a pragmatic approach to sample size estimation was useful, in particular, re-estimation after an adequate run in period, and is recommended where little prior data exists.

## Competing interests

The author(s) declare that they have no competing interests.

## Authors' contributions

SEL wrote the original protocol, secured funding, act as co-principal investigator and lead in the writing of this manuscript. MWC wrote the original protocol, secured funding and act as co-principal investigator. JRD and SW contributed to the development of the protocol and securing funding. JLH and AS contributed to the development of the protocol and securing funding and wrote the statistical and economic aspects of the protocol respectively. RAN is responsible for the day to day co-ordination of the trial, has contributed to refining the protocol and outcome measure package. JLM revised the protocol and the sample size calculations. MC refined the economics section of the protocol and is providing economic input. EJW is responsible for the data management and trial support. All authors have contributed to and approved the final manuscript.

## CAST research team

Lamb SE, Cooke MW, Nakash RA, Withers EJ, Hutton JL, Marsh JL, Szczepura A, Wilson S, Clark M, Dale JR, MacNamara A, Kendall J, Skinner D, Sakr M, Kelly C, O'Byrne G, Morrell R, Dunn M, Bateman S, Kempson S, Hooker F, Parker N, Noakes E, Vaux N, Doughty G, Nichols V, Ritchie C, Sabine E.

### Trial Steering Committee

Professor Bill Gillespie, Professor Sallie Lamb, Dr Matthew Cooke, Professor Jeremy Dale, Professor Jane Hutton, Dr Jen Marsh, Professor Ala Szczepura, Dr Sue Wilson, Rachel Nakash, Vicki Staples.

### Data Monitoring Committee

Dr Janet Dunn, Professor Damian Griffin, Pat Overton-Brown.

## Pre-publication history

The pre-publication history for this paper can be accessed here:


